# Na_3_Ge_2_P_3_: A Zintl Phase Featuring [P_3_Ge–GeP_3_] Dimers as Building Blocks

**DOI:** 10.1021/acs.inorgchem.4c00287

**Published:** 2024-04-19

**Authors:** Manuel Botta, Sabine Zeitz, Wilhelm Klein, Gabriele Raudaschl-Sieber, Thomas F. Fässler

**Affiliations:** † Technical University of Munich (TUM), TUM School of Natural Sciences, Department of Chemistry, Chair of Inorganic Chemistry with Focus on New Materials, Lichtenbergstrasse 4, D-85748 Garching, Germany; ‡ Technical University of Munich (TUM), TUM School of Natural Sciences, Department of Chemistry, Chair of Inorganic and Metal−Organic Chemistry, Lichtenbergstrasse 4, D-85748 Garching, Germany

## Abstract

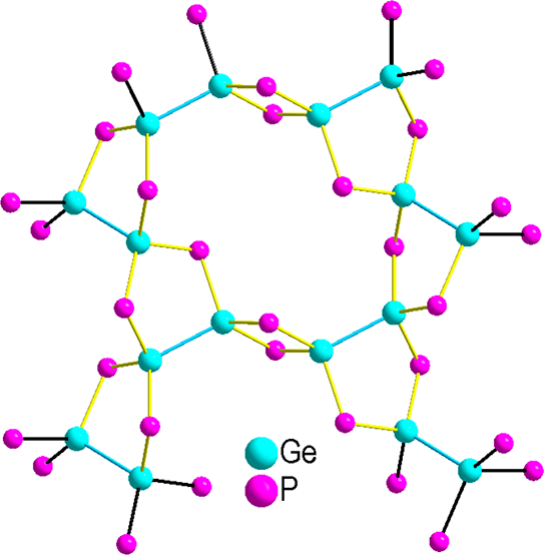

Recently, ternary lithium phosphidotetrelates have attracted interest particularly due to their high ionic conductivities, while corresponding sodium and heavier alkali metal compounds have been less investigated. Hence, we report the synthesis and characterization of the novel ternary sodium phosphidogermanate Na_3_Ge_2_P_3_, which is readily accessible via ball milling of the elements and subsequent annealing. According to single crystal X-ray structure determination, Na_3_Ge_2_P_3_ crystallizes in the monoclinic space group *P*2_1_/*c* (no. 14.) with unit cell parameters of *a* = 7.2894(6) Å, *b* = 14.7725(8) Å, *c* = 7.0528(6) Å, β = 106.331(6)° and forms an unprecedented two-dimensional polyanionic network in the *b/c* plane of interconnected [P_3_Ge–GeP_3_] building units. The system can also be interpreted as differently sized ring structures that interconnect and form a two-dimensional network. A comparison with related ternary compounds from the corresponding phase system as well as with the binary compound GeP shows that the polyanionic network of Na_3_Ge_2_P_3_ resembles an intermediate step between highly condensed cages and discrete polyanions, which highlights the structural variety of phosphidogermanates. The structure is confirmed by ^23^Na- and ^31^P-MAS NMR measurements and Raman spectroscopy. Computational investigation of the electronic structure reveals that Na_3_Ge_2_P_3_ is an indirect band gap semiconductor with a band gap of 2.9 eV.

## Abstract 2

Alkali-metal-containing phosphidotetrelates have recently been shown to be particularly interesting due to their ionic conductivities. Utilizing an accessible and convenient synthesis procedure, we report on the newest compound Na_3_Ge_2_P_3_ in this system. The structure reveals an unprecedented two-dimensional polyanionic network comprising homo- and heteroatomic Ge−Ge and Ge−P bonds, respectively. Computational investigations reveal semiconducting properties as expected for a Zintl phase.

## Introduction

Binary and ternary tetrel pnictides reveal great structural variety and highly interesting properties. For example, many ternary tetrel pnictides exhibit a noncentrosymmetric structure and, together with a suitable band gap, a high laser threshold and good chemical stability, which allows their application as nonlinear optical material.^1−3^ Indeed, in the pnictide system, chalcopyrites^4−7^ were intensively investigated in the late 20th century when they were considered as a benchmarking system for nonlinear optical crystals. More recently found compounds such as MgSiAs_2_^8^ or Ba_2_Si_3_P_6_^9^ even promise to outperform the current standards. Recently, an unexpected thermal behavior was found by incorporating Li or Na into SnPn layers^10−12^ while the compounds Na_1–*x*_Sn_2_P_2_ and NaSn_2_As_2_ are superconducting agents at 2.0 and 1.3 K, respectively.^11,13^ Furthermore, materials with direct band gaps allow for application in optoelectronics, batteries, thermoelectrics, and photovoltaics.^14−16^ However, despite the numerous promising properties and a vast field of applications, pnictides are generally much less investigated than the neighboring system of chalcogenides.^17−20^

The combination of elements from the 14th and 15th group with alkali metals has led to the discovery of several new materials in recent years of which some got alot of attention due to their high superionic conductivity.^21−23^ These ternary phases exhibit a broad structural variety that is highly dependent on the content of alkali metal present. Alkali-rich compounds such as Li_9_TrP_4_,^21,24^ Li_8_TtP_4_^25−27^ or Li_14_TtP_6_^22,23^ show isolated [TtP_4_]^8–^ and [TrP_4_]^9–^ tetrahedra (Tt = Si, Ge, Sn; Tr = Al, Ga) within a *ccp* or *hcp* of phosphorus atoms. In less alkali-rich compounds, the tetrahedra are more condensed and a huge variety of interconnected structure types or networks is formed. These networks range from simple edge-sharing double tetrahedra in Li_10_Si_2_P_6_^28^ and Na_10_Tt_2_P_6_^29,30^ (Tt = Si, Ge, Sn) to complex motifs such as infinite chains of edge-sharing tetrahedra in Na_2_SiP_2_,^31^ layered structures with either exclusively corner or corner and edge-sharing tetrahedra in Li_3_Si_3_P_7_^28^ or Li_3_TrP_2_^32^ (Tr = Al, Ga), respectively, and supertetrahedral structures in Li_2_SiP_2_, LiSi_2_P_3_,^33^ Li_2_GeP_2_, LiGe_3_P_3_,^34^ or Li_3_InP_2_.^35^

Just recently, two new compounds, Na_2_Ge_3_P_3_ and Na_5_Ge_7_P_5_, in the system of sodium phosphidogermanates were reported.^36^ Both compounds feature a complex polyanionic framework including homoatomic Ge–Ge bonds. A structural comparison of these compounds with previously reported NaGe_3_P_3_,^37^ that shows a similar 2D polyanionic structure, or Na_10_Ge_2_P_6_,^30^ which features isolated dimeric building units, reveals that the Na–Ge–P system is very promising to establish a broad variety of possible connection patterns. Moreover, a comparison of these ternary phases with A/Ge binary phases (A = Na, K) featuring polyhedral structure motifs such as in [Ge_4_]^4–^ or [Ge_9_]^4–^ Zintl clusters^38^ or clathrate compounds^39^ hints for a plethora of possible structure motifs.

Thus, we focused on the search for new ternary compounds in this system and report on the preparation of Na_3_Ge_2_P_3_ via ball milling and subsequent annealing of the reactive mixture. The structure comprises [P_3_Ge–GeP_3_] building units which can be interpreted as two interpenetrating Ge(GeP_3_) tetrahedra featuring a homoatomic Ge–Ge bond beside Ge–P bonds. Experimental and calculated Raman investigations coherently show that the most intensive mode is connected to a vibration of the Ge–Ge unit. Furthermore, we investigated the electronic structure with theoretical methods and revealed that Na_3_Ge_2_P_3_ is an indirect band gap semiconductor.

## Experimental Section

All steps of synthesis and sample preparation were carried out inside an argon-filled glovebox (MBraun, *p*(H_2_O), *p*(O_2_) < 0,1 ppm). Prior to use, sodium (Rods, Merck-Schuchardt, > 99%) was cleaned from oxide layers. Germanium (EVOCHEM GmbH, 99,999%) and red phosphorus (powder, Sigma-Aldrich, 97%) were used without any further purification.

### Synthesis of Na_3_Ge_2_P_3_

Na_3_Ge_2_P_3_ was synthesized from the elements via ball milling and subsequent annealing. Sodium (561.4 mg, 24.42 mmol, 3.0 equiv), germanium (1182.3 mg, 16.28 mmol, 2.0 equiv), and red phosphorus (779.7 mg, 25.17 mmol, 3.0 equiv) were added to a WC milling jar (50 mL with three balls with a diameter of 15 mm each) at inert atmosphere. The jar was sealed and transferred to a planetary mill (Retsch PM 100) where it was ball-milled for 18 h at 350 rpm in intervals of 10 min with direction reversal and subsequent 5 min resting. The obtained black powder which appeared with reddish stroke color was added to niobium tubes (8 mm diameter) in batches of 200 mg, sealed in an electric arc furnace (Edmund Bühler MAM1), enclosed in a silica reaction container under vacuum, subsequently heated with 5 K·min^–1^ up to 873 K, and dwelled for 48 h in a tube furnace (HTM Reetz Loba 1200-42-600-1-OW with a EUROTHERM S 14083 temperature controller) and cooled at a rate of 0.5 K·min^–1^. After grinding, a black powder was obtained.

Black, shiny single crystals were obtained from a sample with the nominal composition of “Na_2_GeP_2_”. Herein an equivalent two-step synthesis as above was carried out starting with the elements sodium (636.63 mg, 27,69 mmol, 2.0 equiv), germanium (1005.64 mg, 13.85 mmol, 1.0 equiv), and phosphorus (884.25 mg, 28.55 mmol, 2.0 equiv). Instead of niobium tubes, carbon-coated quartz glass ampules were used.

### Powder X-ray Diffraction and Rietveld Refinement

For powder X-ray diffraction (PXRD) measurements, the samples were ground in an agate mortar and sealed inside 0.3 mm glass capillaries. PXRD measurements were performed at room temperature on a Stadi P diffractometer (Stoe & Cie) equipped with a Ge(111) monochromator for Cu K_α1_ radiation (λ = 1.5406 Å) and a MYTHEN DCS 1K (Dectris) solid-state detector. The raw powder data were processed with the software package WinXPOW.^40^ Rietveld refinement was executed with the FullProf program package.^41^ Single crystal data from the structure determination were used as the structural model. To model the peak profile shape, the pseudo-Voigt function was chosen. Background contribution was determined using a linear interpolation between selected data points in nonoverlapping regions. The scale factor, zero angular shift, profile shape parameters, resolution (Caglioti) parameters, and asymmetry and lattice parameters as well as fractional coordinates of atoms and their isotropic displacement parameters were varied during the fitting.

### Single Crystal Structure Determination

A single crystal of Na_3_Ge_2_P_3_ was sealed in a 0.3 mm glass capillary. The single crystal X-ray diffraction (SCXRD) measurement was performed on a StadiVari diffractometer (Stoe & Cie) equipped with a Ge(111) monochromator, a Mo K_α1_ radiation (λ = 0.71073 Å) source and a PILATUS 3R 300 K detector (Dectris). The structure was solved by Direct Methods (SHELXS) and refined by full-matrix least-squares calculations against *F*^2^ (SHELXL).^42^ Further details of the crystal structure investigations may be obtained from the joint CCDC/FIZ Karlsruhe online deposition service: https://www.ccdc.cam.ac.uk/structures/ by quoting the deposition number CSD-2286066 (single crystal data) and CSD-2286067 (powder data).

### Differential Scanning Calorimetry (DSC)

For thermal analysis, 50 mg of Na_3_Ge_2_P_3_ was added to a niobium ampule (2 mm diameter), sealed by arc welding, and measured on a DSC machine (Netzsch, DSC 404 Pegasus) under a constant gas flow of 75 mL·min^–1^. The sample was heated to 1023 K and cooled to 423 K twice at a rate of 10 K·min^–1^. To determine the onset temperatures of the DSC signals, the PROTEUS Thermal Analysis software was used.^43^

### Raman Spectroscopy

Raman spectra were measured using an inVia Raman microscope (Renishaw, RE04), equipped with a CCD detector. The powdered sample was sealed into a 0.3 mm glass capillary and irradiated with a 785 nm laser beam for 1 s at 0.1% laser power using a microscope equipped with a 50-fold magnifying objective and a grating with 1800 lines mm^–1^. For the final spectrum, 100 single measurements were averaged. The software WiRe 4.2 (build 5037, Renishaw 2002) was used for data recording.^44^

### NMR Spectroscopy

Magic angle spinning (MAS) NMR spectra were recorded on a Bruker Avance 300 NMR device operating at 7.04 T in a 4 mm ZrO_2_ rotor. The resonance frequencies of the nuclei were 79.39 and 121.46 MHz for ^23^Na and ^31^P, respectively. The rotational frequency was set to 15 kHz for all nuclei. The MAS spectra were obtained at room temperature with relaxation delays of 10 s (^23^Na) and 30 s (^31^P) and 880 (^23^Na) and 2340 scans (^31^P). The ^23^Na spectrum is referenced to NaCl (1 M, aq) and NaCl (s) with chemical shifts of 0.00 ppm and +7.20 ppm, respectively. The ^31^P spectrum is referred to ammonium dihydrogen phosphate (s) with a chemical shift of 1.11 ppm with reference to concentrated H_3_PO_4_. All spectra were recorded using single-pulse excitation.

### Electronic Structure Calculations

The computational analysis of Na_3_Ge_2_P_3_ was performed using the Crystal17 program package and hybrid density functional methods.^45,46^ A hybrid exchange correlation functional after Perdew, Burke, and Ernzerhof (PBE0)^47,48^ and triple-ζ valence + polarization level basis sets for Ge and P and split valence + polarization for Na derived from the Karlsruhe basis sets were applied (further details are given in the Supporting Information).^49−52^ The starting geometry was taken from the experimental data, and the structure was fully optimized within the constraints imposed by the space group symmetry. Band structure and density of states (DOS) were calculated. The Brillouin zone path of Na_3_Ge_2_P_3_, Γ-Z-D-B-Γ-A-E-Z-C_2_-Y_2_-Γ was provided by the web application *SeeK-path*.^53^ The nature of a stationary point on the potential energy surface was confirmed to be a minimum by a frequency calculation at Γ-point. No imaginary frequencies were observed.

## Results and Discussion

### Synthesis and Structure of Na_3_Ge_2_P_3_

**Figure 1:**
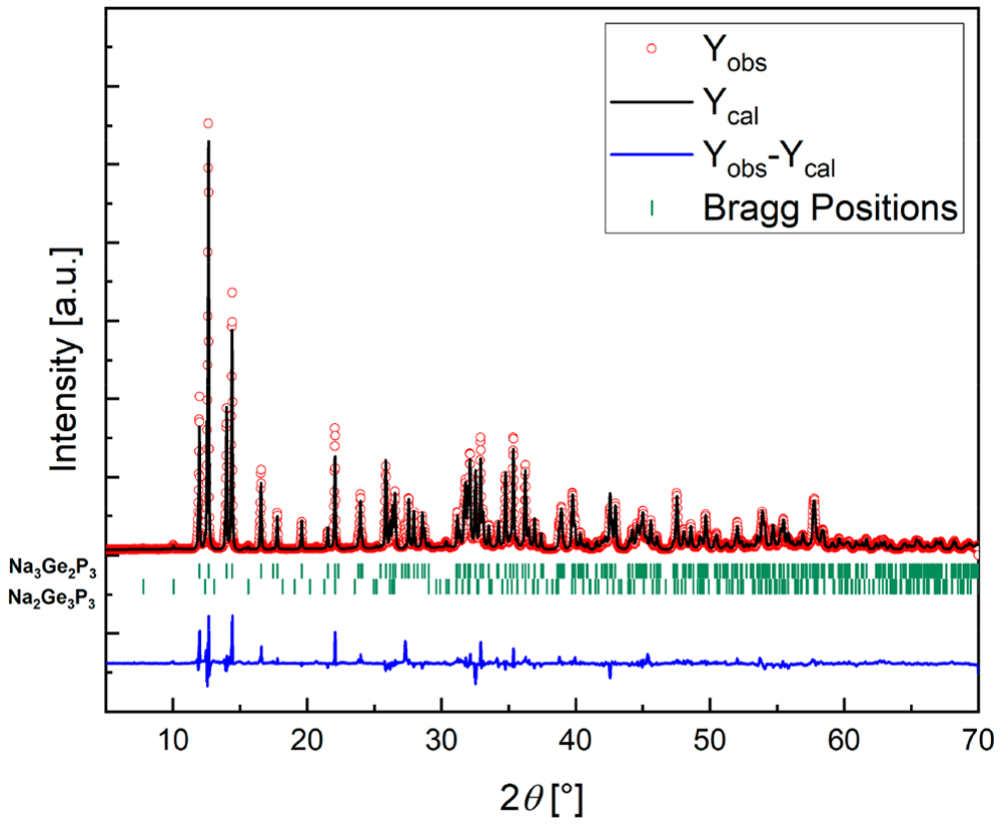
Rietveld refinement of the powder X-ray pattern of Na_3_Ge_2_P_3_. The red line indicates observed intensities, the black line indicates the calculated intensities, and the blue line shows the difference. Bragg positions are depicted as green dashes. The Na_3_Ge_2_P_3_:Na_2_Ge_3_P_3_ ratio is 98.7(4) wt %:1.2(1) wt %, respectively.

During the search for new compounds in the system of sodium phosphidogermanates, the compound Na_3_Ge_2_P_3_ was synthesized from the elements via a two-step procedure. First, stoichiometric amounts of sodium, germanium, and red phosphorus were ball-milled to form a reactive mixture which, according to X-ray powder diffraction, is mainly amorphous with significant traces of elemental germanium. Subsequently, the reactive mixture was annealed in a niobium tube at 600 °C for 2 days. Na_3_Ge_2_P_3_ is obtained as a black powder with minor impurities of Na_2_Ge_3_P_3_. The Na_3_Ge_2_P_3_:Na_2_Ge_3_P_3_ ratio is 98.7(4) wt %:1.2(1) wt % as determined by Rietveld refinement (Figure 1).

**Figure 2:**
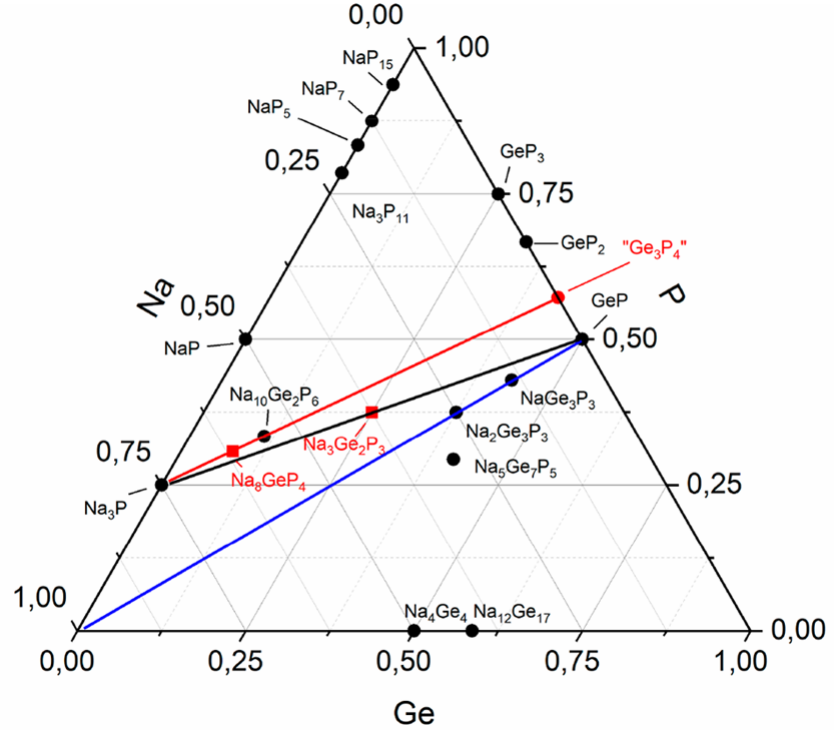
Ternary Na–Ge–P composition diagram. Red, black, and blue lines indicate the imaginary lines between Na_3_P and hypothetical “Ge_3_P_4_”, between Na_3_P and GeP and between Na and GeP, respectively.

DSC measurements up to 800 °C show several smaller signals, probably indicating several phase formation and transformation phenomena in this phase composition (Figure S5). The composition of the title compound matches the imaginary line of Na_3_P and GeP in the Gibbs composition triangle (Na_3_Ge_2_P_3_ = Na_3_P + 2 GeP, Figure 2) and marks the first known compound in this line. Complete data of the Rietveld refinement and additional powder patterns are given in the Supporting Information (Figures S1 and S2 and Table S5).

Black, shiny single crystals were obtained from a sample with the nominal composition of “Na_2_GeP_2_” which was annealed at 600 °C in carbon-coated silica-glass ampules. The details of single crystal X-ray structure refinement data of Na_3_Ge_2_P_3_ are listed in Table 1.

**Table 1:** Crystallographic Refinement Data of Single Crystal Diffraction on Na_3_Ge_2_P_3_ at 293 K

sum formula	Na_3_Ge_2_P_3_
molar mass/g mol^–1^	307.06
crystal system	monoclinic
space group	*P*2_1_*/c* (14)
radiation λ/Å	0.71073 (Mo K_α_)
crystal size/mm^3^	0.15 × 0.10 × 0.10
crystal color	black
cell parameters/Å	*a* = 7.2894(6)
	*b* = 14.7725(8)
	*c* = 7.0528(6)
	β = 106.331(6)°
cell volume/Å^3^	728.82(10)
*Z*	4
ρ (calcd)/g cm^–3^	2.798
μ/mm^–1^	8.967
*F*(000)	568
temperature/K	293(2)
reflections collected	18774
independent reflections	2547
reflections with *I* > 2*σ*(*I*)	1876
observed *hkl*	–11 ≤ *h* ≤ 10
	–22 ≤ *k* ≤ 22
	–10 ≤ *l* ≤ 10
measuring range θ_min*/*max_/deg	2.91/32.82
goodness-of-fit on *F*_o_^*2*^	0.933
*R*_int_*/R*_*σ*_	0.0549/0.0501
*R* indices (*F*_0_^*2*^ ≤ 2*σ*(*F*_0_^*2*^))	*R*1 = 0.033, *wR*2 = 0.060
*R* indices (all data)	*R*1 = 0.059, *wR*2 = 0.066
largest diff peak/hole/e^–^ Å^–3^	1.373/–1.140
depository no.	CSD-2286066

**Figure 3:**
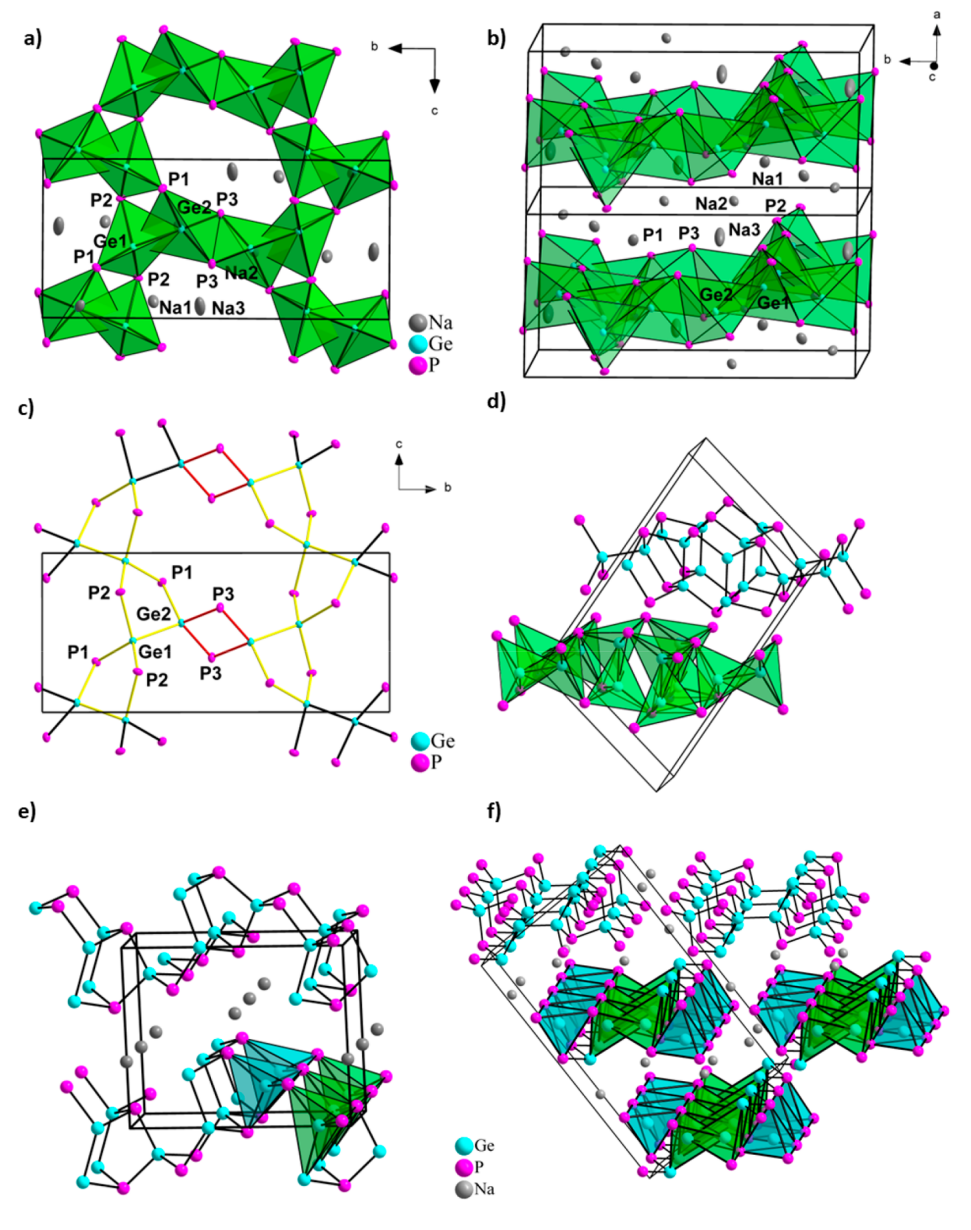
Crystal structure of Na_3_Ge_2_P_3_. (a) View along [100] onto the extended unit cell. The [P_3_Ge–GeP_3_] subunit of two interpenetrated tetrahedra is connected by one edge and four corners to neighboring groups. The subunit is highlighted with green polyhedra centered by Ge atoms. (b) Two-dimensional layers of polyanions of Na_3_Ge_2_P_3_ in the *b*/*c* plane. (c) As Figure 3a with emphasis on the atom connection revealing five-membered Ge_3_P_2_ rings depicted in yellow. Planar four membered Ge_2_P_2_ rings connecting the parallel chains are depicted in red. The Na, Ge, and P atoms are drawn in gray, blue, and pink, respectively, and displacement ellipsoids are shown at a 70*%* probability level. (d) Structure of GeP^56^ along the *b*-axis. Similar [P_3_Ge–GeP_3_] building units can be identified and are depicted in green. Extended unit cells of (e) NaGe_3_P_3_^37^ and (f) Na_2_Ge_3_P_3_,^36^ showing the respective anionic GeP substructure with covalent bonds in the upper and tetrahedra around Ge atoms in the lower part. Units with Ge–Ge bond similar to the [P_3_Ge–GeP_3_] unit are depicted in green, and GeP_4_ units are depicted in turquoise. Na, Ge, and P atoms are drawn in gray, blue, and pink, respectively.

The compound Na_3_Ge_2_P_3_ crystallizes as black chunks in the monoclinic space group *P*2_1_/*c* (no. 14.) in a novel structure type. The crystal structure contains eight crystallographically independent positions, three sodium (Na1–Na3), two germanium (Ge1 and Ge2), and three phosphorus sites (P1–P3), all at general 4*e* Wyckoff positions. Ge and P are covalently connected forming an anionic Ge/P partial structure, which is surrounded by Na^+^ ions. The structural motif is a repeating ethane-like Ge_2_P_6_ dimeric unit in staggered conformation which are connected via common P atoms to neighboring units. The characteristic tetrahedral coordination of the Ge atoms which has been observed in other ternary tetrel phosphides is conserved here but with one P vertex per tetrahedron substituted by Ge. Therefore, the [P_3_Ge–GeP_3_] dimeric-unit can be interpreted as two interpenetrated and crystallographically independent Ge(GeP_3_) tetrahedra (Ge1 surrounded by Ge2, P1, P2, and P2′ as well as Ge2 surrounded by Ge1, P1, P3, and P3′) where each utilizes the central germanium atom of the neighboring tetrahedron as a corner atom as shown in Figure 3. These subunits are connected to neighboring subunits by sharing the P3–P3′ edge of the Ge2 tetrahedron as well as via common P1 and P2 vertices that are bound to Ge2 and Ge1, respectively. In consequence, a two-dimensional porous network in the *b/c*-plane is formed (see Figure 3a and 3b). Considering covalent bonds between atoms of p-block elements, the network can also be described as chains of bent Ge_3_P_2_ rings parallel to the *c* axis, which are connected by bridging P3 atoms in *b* direction (Figure 3c). These five-membered rings are composed of two Ge1, one Ge2, one P1, and one P2 atom. Herein each Ge1 atom is part of two rings connecting them at two corners and forming infinite chains of five-membered rings along the *c*-direction, thus forming a spiro-type polyanion. The bridging P3 atoms form planar Ge_2_P_2_ rings together with two Ge2 atoms, completing the two-dimensional network in the *b/c* plane. The Ge–Ge bond length of 2.4885(5) Å is slightly elongated compared to those found in elemental Ge (2.450 Å)^54^ and is in the same range as in structurally related ternary compounds such as NaGe_3_P_3_ (Ge–Ge, 2.626 Å) or Na_2_Ge_3_P_3_ (Ge–Ge, 2.463 Å).^36,37^ Further on, Ge–P bond lengths of 2.3191(9)–2.3607(10) Å in the Ge(GeP_3_) tetrahedra are in good agreement with Ge–P distances found in other compounds of the same ternary system.^30,36,37^ Interestingly, bond angles of 96.04(3)–122.14(3)° show relatively high deviation from the ideal tetrahedron angle of 109.47°. The Na atoms are located in cavities of the two-dimensional polyanions as well as in between the layers. Na1 and Na2 are coordinated by distorted trigonal bipyramids of phosphorus atoms while Na3 is coordinated tetrahedrally but heavily shifted away from the center. Na–P distances range from 2.841(2) to 3.267(2) Å which is in the range of binary Na_3_P^55^ and other known ternary sodium compounds. Na_3_Ge_2_P_3_ is electronically balanced according to the Zintl concept by comprising exclusively four- and two -fold bonded 4b-Ge and 2b-P atoms (nb = n-fold bonded atom), respectively, and a formal electron transfer from the Na atoms to the polyanionic framework resulting in the electronically balanced formula (Na^+^)_3_[(4b-Ge)^0^]_2_[(2b-P)^−^]_3_ including covalent Ge–Ge and Ge–P bonds.

### Comparison of the Structures Na_3_Ge_2_P_3_, GeP, and Related Ternary Na–Ge–P Compounds

The crystal structure of Na_3_Ge_2_P_3_ represents a novel structure type with a unique anionic partial structure and follows a general trend in the ternary Na–Ge–P chemical system of forming surprising and hardly predictable structures. This trend is strikingly different from the Na–Si–P system which exhibits SiP_4_ tetrahedra, either isolated or connected via common P-atoms, as dominant building units in the anionic structure parts. Here, the compounds contain almost exclusively heteroatomic Si–P bonds.^30,31,57^ In the ternary Na–Ge–P system, only two out of six known compounds form anionic partial structures that follow this pattern. While the just recently found Na_8_GeP_4_ consists of isolated [GeP_4_]^8–^ tetrahedral anions,^58^ in Na_10_Ge_2_P_6_ two GeP_4_ tetrahedra are connected via a common edge forming isolated [Ge_2_P_6_]^2–^ anions with four terminal and two bridging P atoms.^30^ Notably, these two compounds are found on the line between Na_3_P and the hypothetical “Ge_3_P_4_” in the corresponding Gibbs composition triangle of Na, Ge, and P and are derived by the linear combination of Na_3_P and Ge_3_P_4_: 1 × Ge_3_P_4_ + 5 Na_3_P = 3 Na_5_GeP_3_ or 2.5 × Na_10_Ge_2_P_6_ as well as 1 × Ge_3_P_4_ + 8 Na_3_P = 3 Na_8_GeP_4_. “Ge_3_P_4_” is predicted to adapt the structure of the lighter homologue Si_3_N_4_ comprising exclusively 4b-Ge and 3b-P atoms as well as solely Ge–P heteroatomic bonds^59,60^ which is in accordance with the two reported ternary examples on the line.

Close similarities with the here-presented Na_3_Ge_2_P_3_ are found in the binary compound GeP.^56^ Here, each Ge atom forms one Ge–Ge and three Ge–P bonds while all P atoms are connected to three Ge atoms. The resulting Ge(GeP_3_) tetrahedra are interpenetrating each other, as the center is one corner of the neighboring one, together forming a [P_3_Ge–GeP_3_] unit which is connected via common P atoms to neighboring units. The resulting two-dimensionally infinite layers (Figure 3d) have the phosphorus lone pairs pointing toward the gap between the layers, representing a similar connectivity of the atoms as found in Na_3_Ge_2_P_3_. Since Na_3_Ge_2_P_3_ fits on the line connecting Na_3_P and GeP it retains their bond characteristic and can be written as a linear combination of both binaries according to Na_3_P + 2 GeP = Na_3_Ge_2_P_3_.

The compounds NaGe_3_P_3_^37^ and Na_2_Ge_3_P_3_^36^ are located on the line from Na and GeP, corresponding to the addition of one and two Na equivalents to three GeP units, respectively. These compounds are all connected by bearing homoatomic Ge–Ge bonds as the common structural feature. In NaGe_3_P_3_^37^ the anionic ^2^_∞_[Ge_3_P_3_]^−^ substructure includes GeP_4_ tetrahedra while retaining Ge–Ge bonds and including (3b-Ge)^1–^ atoms that formally bear a negative charge (Figure 3e). Interestingly, in this structure, all P atoms, although marginally more electronegative, form three covalent bonds and have no formal charge. The main building blocks forming the anionic partial structure are composed of five- and six-membered rings representing a heteroatomic variation of sections of Hittorf’s phosphorus.^61^ Formal addition of two Na results in Na_2_Ge_3_P_3_ where the two-dimensional anionic partial structure is cleaved into one-dimensional ^1^_∞_[Ge_3_P_3_]^2–^ strands.^36^ In detail, the [Ge_3_P_3_] building blocks from NaGe_3_P_3_ are maintained but show different connection, as Ge–Ge dimers are now connected forming Ge_4_ units and a (2b-P)^1–^ bridge adds an additional negative charge to the polyanion (Figure 3f). Here, the anionic partial structure can be described as strands of corner-sharing GeP_4_ tetrahedra which are connected by rows of parallel Ge_4_ chains. Another compound with the same connection pattern but different composition, namely Na_5_Ge_7_P_5_,^36^ results from a partial substitution of a P atom by Ge which extends the Ge_4_ chain and adds another Na atom position. Thus, the compound does not appear on one of the lines of the composition diagram described above. Compared to Na_3_Ge_2_P_3_, the Ge content is slightly lowered, so the aggregation of Ge atoms is also reduced and only Ge_2_ units are found.

### MAS NMR and Raman Spectroscopy

**Figure 4:**
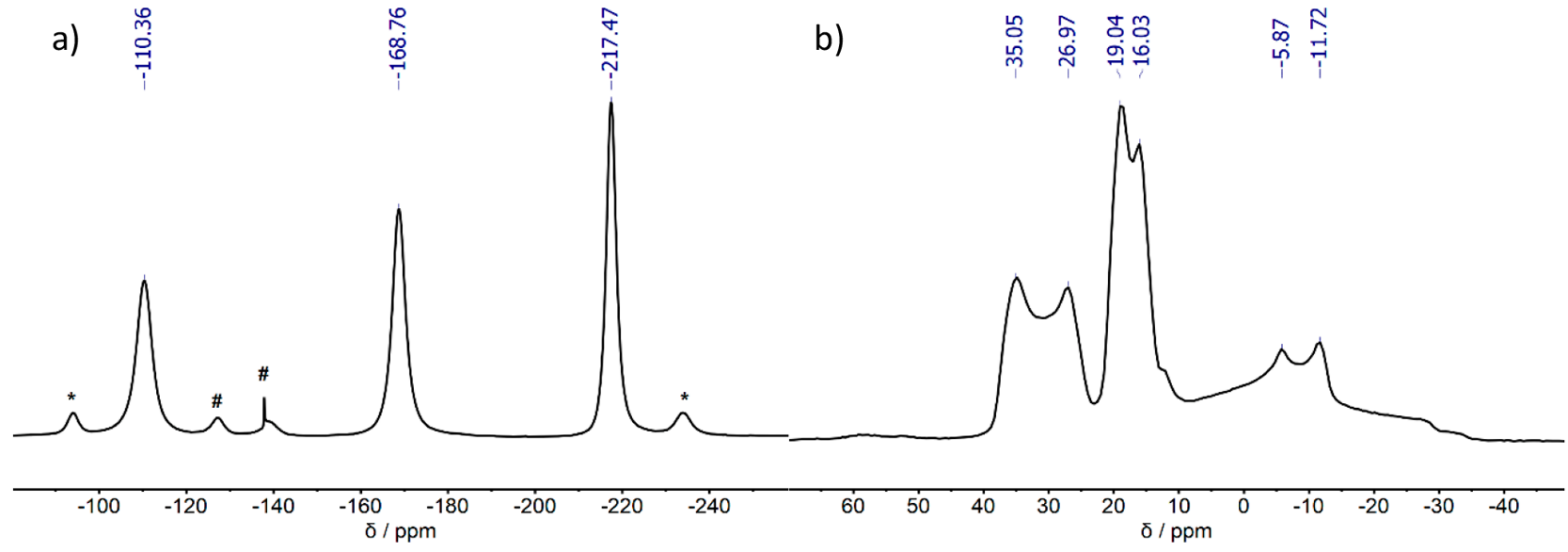
(a) ^31^P and (b) ^23^Na MAS NMR spectra of Na_3_Ge_2_P_3_. The ^31^P NMR spectrum shows three signals with an approximate ratio of 2:3:4. The ^23^Na NMR spectrum shows three doublet signals. Chemical shifts are indicated as numbers above each signal. Rotational sidebands are marked with *. Signals from the side phase Na_2_Ge_3_P_3_ are marked with #.

The ^31^P MAS NMR spectrum of Na_3_Ge_2_P_3_ (Figure 4) shows three sharp signals at −110.4, −168.8, and −217.5 ppm. Several smaller signals (* and #) are assigned as rotational sidebands of the main signals and as signals from the sidephase Na_2_Ge_3_P_3,_ respectively. The integrals of the signals with their matching rotational sidebands considered result in a ratio of 1:1:1 matching the observed three crystallographic phosphorus positions. The range of the observed signals (−110 to −217 ppm) matches the chemical shift regime of (2b-P)^1–^ observed in compounds such as Li_3_Si_3_P_7_^28^ (−168.8 and −178.7 ppm) and Li_2_SiP_2_^27^ (−129.1 and −241.5 ppm). As an increased downfield shift can be interpreted as higher negative charge on the phosphorus site, the P3 atoms, which possess the longest Ge–P distances where the tetrahedra are connected via egde-sharing, are assigned to the signal at δ = −217.5 ppm. The other two phosphorus positions, which object to the corner sharing edges of the tetrahedra, are assigned to the signals at δ = −110.4 and −168.8 ppm.

The ^23^Na MAS NMR spectrum shows three broad, slightly overlapping but distinguishable doublet signals in the range of −40 to 40 ppm. The three signals can be integrated to a value of one each and therefore be assigned to the three crystallographic Na positions.

**Figure 5:**
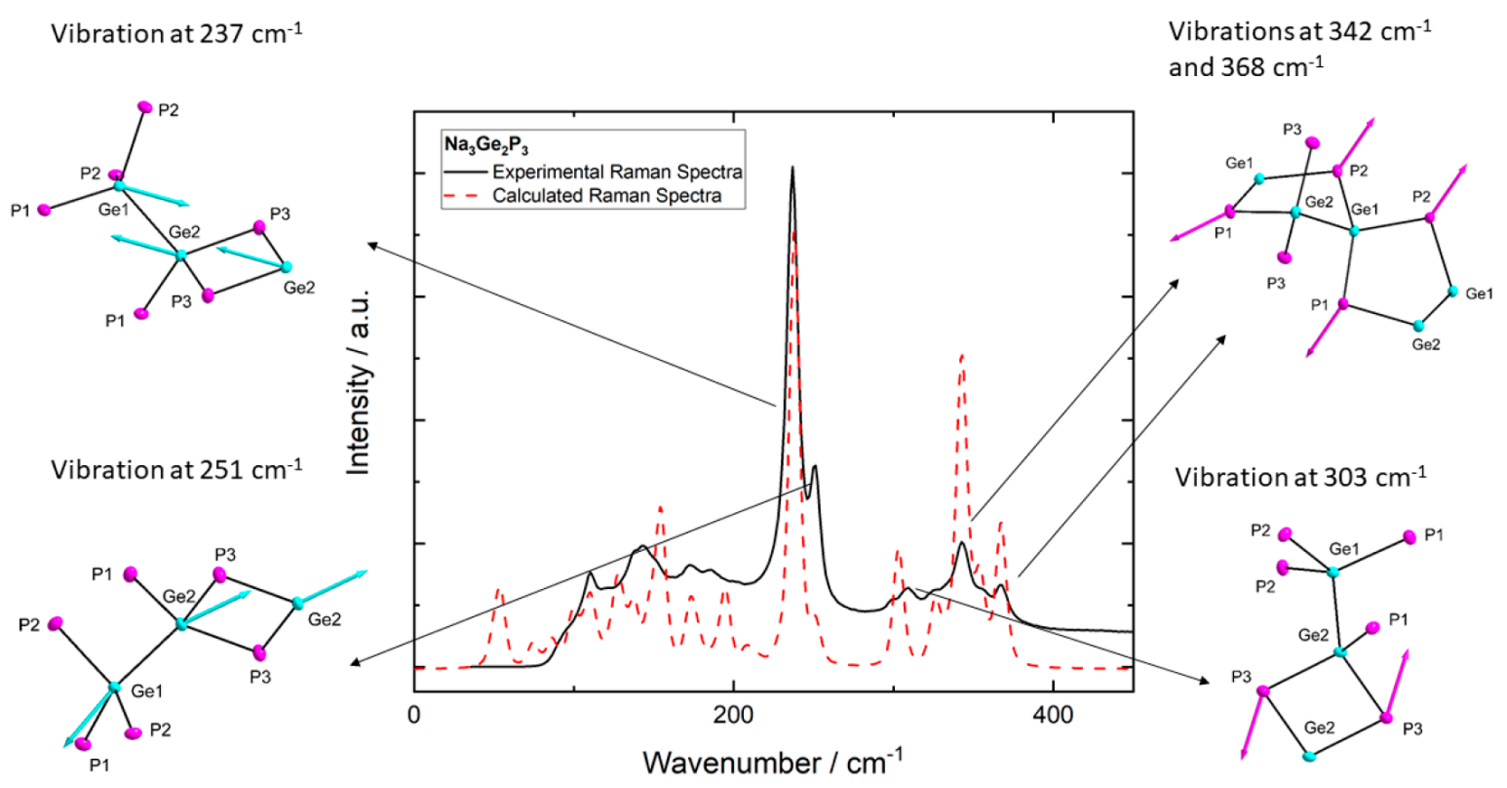
Calculated (black) and experimental (red) Raman spectra of Na_3_Ge_2_P_3_. Vibrations at 237 and 251 cm^–1^ represent two different asymmetric bending/stretching modes of the Ge–Ge bond. Vibrations at 303 cm^–1^ represent the bending modes of the P3 atoms. Vibrations at 342 and 368 cm^–1^ represent the asymmetric stretching of P1 and P2 atoms, respectively. The wavenumbers of the calculated Raman spectrum were scaled by a factor of 0.95 to match the highest intensity of the experimental spectrum.

The Raman spectrum of a powdered sample shows five distinguishable signals with two in the range of 230–250 cm^–1^ and three between 303 and 370 cm^–1^ (Figure 5), with additional signals of lower intensity in the region of 100–200 cm^–1^. For differentiation and assignment to vibrational modes, a Raman spectrum was calculated by means of quantum chemical methods at a DFT-PBE0/TZVP (only SVP for Na) level of theory. The experimental and calculated spectra show good agreement except for slightly shifted wavenumbers. To account for this calculated values were scaled by a factor of 0.95 to compensate the overestimation of the calculation at high wave numbers. The strongest experimentally observed signals are at 237 and 251 cm^–1^ and can be assigned to two asymmetric stretching modes of the Ge–Ge bond. The observed wavenumbers are in good agreement with known strong and characteristic Raman shifts of Ge–Ge “breathing” modes found in [Ge_4_]^4–^ (274 cm^–1^) and [Ge_9_]^4–^ (222 cm^–1^) clusters^39^ as well as in elemental Ge and Si–Ge alloys.^65,66^ The signals observed at higher Raman shifts represent different asymmetric vibrational modes of Ge–P bonds. Naturally due to the two-bonded situation of each phosphorus atom, all vibrational modes are coupled to vibration of neighboring atoms and conclusively to the whole network. The signal at 303 cm^–1^ can be assigned to an asymmetric bending mode of the P3 atom bridges connecting the chains of five-membered rings. This coherently suggests an antiparallel vibration of the infinite chains. The signals at 342 and 368 cm^–1^ are assigned to asymmetric stretching of the P1 and P2 atoms, respectively. The observed asymmetry is correlated to the two-bonded situation of the phosphorus. Furthermore, all signals assigned to phosphorus vibrations match the observed signals found for example in isolated TaP_4_ tetrahedra.^67^

### Investigation of the Electronic Properties of Na_3_Ge_2_P_3_

**Figure 6:**
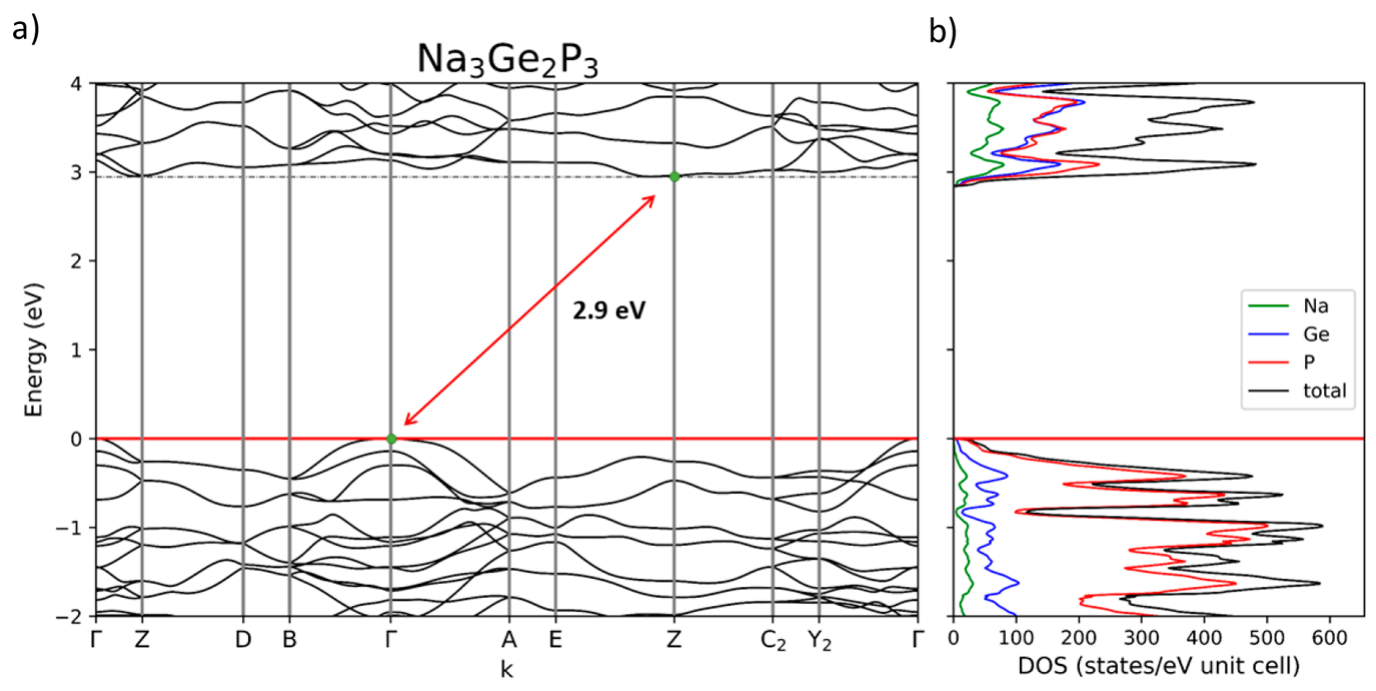
Band structure (a) and density of states (b) of Na_3_Ge_2_P_3_. It reveals an indirect band gap of 2.9 eV and a direct band gap of 3.0 eV at **Γ**. The Fermi level is located at 0 eV.

The electronic property calculations were performed using quantum chemical methods at a DFT-PBE0/TZVP (SVP for Na) level of theory. Experimental crystallographic data were used as starting point for structure optimization. Results are reasonable, as the calculated structure is in line with the experimental one with a maximum deviation of 1% at cell parameter *a*. The electronic structure calculation reveals that Na_3_Ge_2_P_3_ is a semiconductor with an indirect bandgap of 2.9 eV and an about 0.1 eV larger direct band gap at **Γ**. Both the calculated densities of states and the electronic structure are shown in Figure 6a and 6b, respectively. The DOS reveals that phosphorus has the main contribution to the valence bands while germanium shows only weak and sodium barely any contribution. The contribution to the conduction band is different, as germanium shows a similarly high contribution as phosphorus here. The only other electronically investigated sodium phosphidogermanate, NaGe_3_P_3_, exhibits a direct band gap of 2.0 eV.^37^

## Conclusions

The phosphidogermanate Na_3_Ge_2_P_3_ can be readily synthesized via ball-milling and subsequent annealing at 600 °C. The crystal structure features dimeric [P_3_Ge–GeP_3_] building units of interpenetrated Ge(GeP_3_) tetrahedra that form a two-dimensional polyanionic network which is related to the framework found in the binary compound GeP. The connection pattern containing a mixture of corner and edge-sharing [P_3_Ge–GeP_3_] subunits leads to the formation of a system composed of four- and five-membered rings. In this way three-dimensional pathways for the location of counterions are featured in between the layers and through the cavities of the network.

The general trend of reduced dimensionality of the anionic partial structure with increasing content of cations is well-known for Zintl phases. In contrast to the Na–Si–P system, however, several examples are found on the line of the Gibbs composition diagram ending in GeP. As a result, a striking structural feature in the Na/Ge/P system in comparison to the corresponding Na/Si/P system is the occurrence of Ge–Ge bonds and of three-bonded Ge atoms. Ge and P exhibit quite similar electronegativities of 2.0 and 2.2, respectively,^62^ so polar bonds or the localization of a formal negative charge might be less pronounced and homoatomic bonds beside heteroatomic bonds become more favorable.^63,64^ Consequently, further compounds with unusual compositions and different structure motifs may be possible in the Na–Ge–P system. Electronic structure calculations show that the compound is an indirect band semiconductor with a band gap of 2.9 eV where direct and indirect band gap only differ by around 0.1 eV. With this, Na_3_Ge_2_P_3_ demonstrates that ternary phosphidotetrelates are a promising class for tunable direct band gap semiconductors.

## Supporting Information Available

Details on experimental-powder-XRD measurements as well as additional crystallographic data. Experimental data on DSC measurements as well as details on the quantum chemical calculations. The crystallographic data is provided at the joint CCDC/FIZ Karlsruhe online deposition service: https://www.ccdc.cam.ac.uk/structures/ by quoting the deposition number CSD-2286066 (single crystal data) and CSD-2286067 (powder data) (PDF)
