# Supplementary material for: Na3Ge2P3: A Zintl Phase Featuring [P3Ge–GeP3] Dimers as Building Blocks
Source: Inorg Chem. 2024 Apr 19;63(43):20108–16. doi: 10.1021/acs.inorgchem.4c00287 (PMC11523322; doi:10.1021/acs.inorgchem.4c00287)
Supplement: Supplementary file 1 — ic4c00287_si_001.pdf [file ic4c00287_si_001.pdf]

SUPPORTING INFORMATION

$Na_3Ge_2P_3$: A Zintl phase featuring $[P_3Ge\text{-}GeP_3]$ dimers as building blocks

Manuel Botta,[a] Sabine Zeitz,[a] Wilhelm Klein, [a] Gabriele Raudaschl-Sieber,[b] Thomas F. Fässler*[a]

[a] Technical University of Munich (TUM), TUM School of Natural Sciences, Department of Chemistry, Chair of Inorganic Chemistry with Focus on New Materials, Lichtenbergstraße 4, D-85748 Garching, Germany

E-mail: Thomas.faessler@lrz.tum.de

[b] Technical University of Munich (TUM), TUM School of Natural Sciences, Department of Chemistry, Chair of Inorganic and Metal-Organic Chemistry, Lichtenbergstraße 4, D-85748 Garching, Germany

Content:	*page*
Experimental powder X-ray patterns	*S-2*
Results of the Rietveld analysis	*S-3*
Details of the single crystal structure determination	*S-4-5*
Structure of $Na_3Ge_2P_3$	*S-6*
DSC measurement	*S-8*
Results of the quantum chemical calculations	*S-9-12*

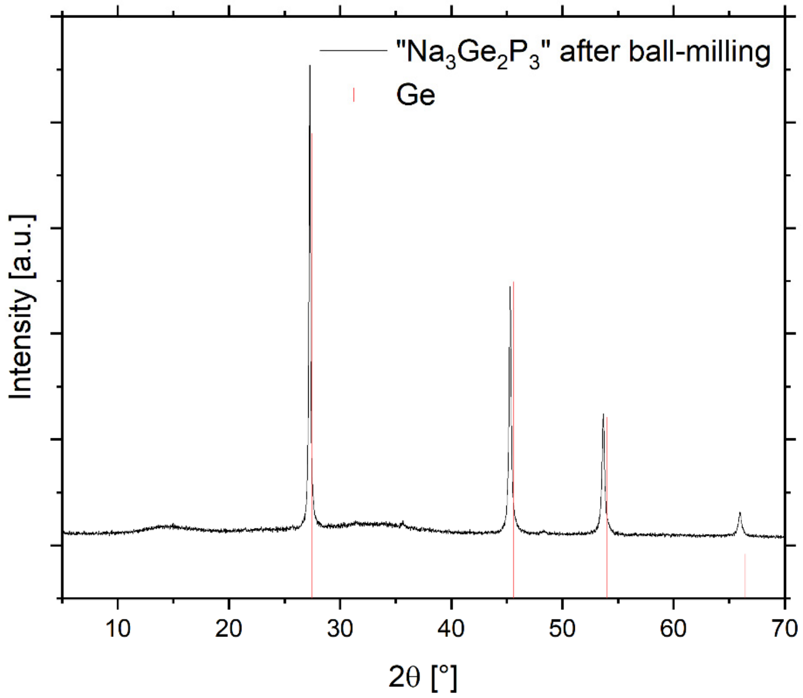

Figure S1. Experimental powder X-ray diffraction pattern of a sample in the stoichiometric ratio of "$Na_3Ge_2P_3$" after ball milling.

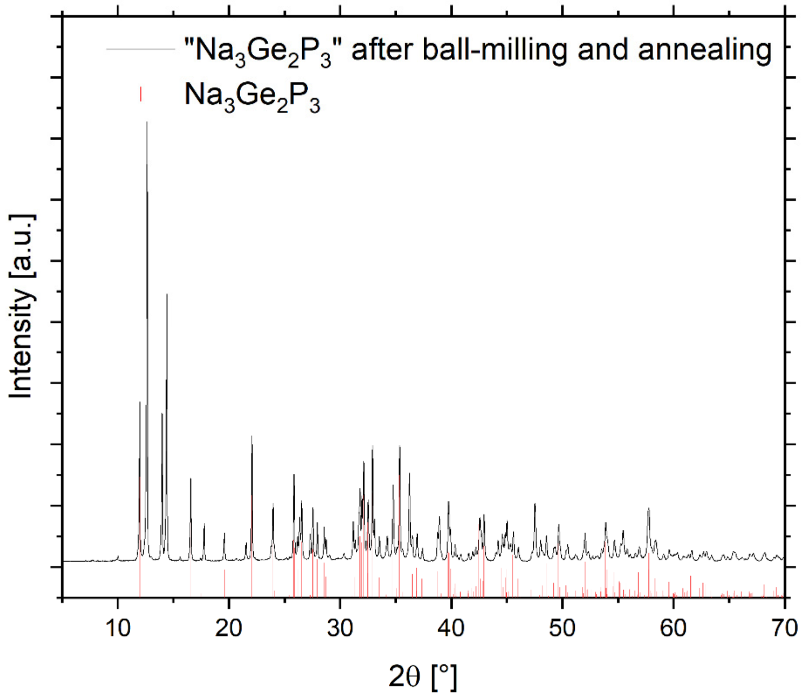

Figure S2. Experimental powder X-ray diffraction pattern of the product a sample in the stoichiometric ratio of "$Na_3Ge_2P_3$" after ball milling and subsequent annealing at 600°C in niobium ampoules

Table S1. Crystallographic data of $Na_3Ge_2P_3$ obtained from powder XRD by Rietveld refinement.

Empirical formula	$Na_3Ge_2P_3$
Formula weight	307.15 g·mol^{-1}
Temperature	293 K
Color	black
Crystal system	monoclinic
Space group	*P21/c* (14)
Lattice parameters	a = 7.28085(6) Å
	b = 14.75274(13) Å
	c = 7.03462(6) Å
	β = 106.3818(7)°
V	724.930(10) Å^3
Z	4
ρ (calc.) / g cm^{-3}	2.81372
Wavelength	1.540600 Å
Θ Range	5.0194° – 69.9993°
R_p	0.073502
R_{wp}	0.102280
R_{exp}	0.022707
X^2	20.2881
Depository no.	CSD-2286067

Table S2. Atomic coordinates and isotropic displacement parameters for $Na_3Ge_2P_3$ from Rietveld analysis.

Atom	Wyckoff pos.	*x/a*	*y/b*	*z/c*	U_{iso} [Å^2]
Na1	4e	0.1704(11)	0.3173(5)	0.1047(12)	0.0127
Na2	4e	0.014(1)	0.3897(5)	0.5763(11)	0.0127
Na3	4e	0.7033(11)	0.0481(5)	0.5833(12)	0.0127
Ge1	4e	0.6318(4)	0.26129(19)	0.4491(4)	0.0127
Ge2	4e	0.5003(4)	0.40076(18)	0.5597(4)	0.0127
P1	4e	0.3893(9)	0.1560(4)	0.3095(10)	0.0127
P2	4e	0.8409(9)	0.2739(4)	0.2512(9)	0.0127
P3	4e	0.2607(9)	0.4885(4)	0.3456(10)	0.0127

Table S3. Atomic coordinates for $Na_3Ge_2P_3$ from a single crystal structure determination at 298 K.

Atom	Wyckoff pos.	*x/a*	*y/b*	*z/c*
Na1	4e	0.1749(3)	0.31989(11)	0.1053(3)
Na2	4e	0.0134(2)	0.39148(11)	0.5759(2)
Na3	4e	0.7082(4)	0.04575(14)	0.5762(4)
Ge1	4e	0.63101(5)	0.26077(2)	0.44882(5)
Ge2	4e	0.49991(5)	0.40029(2)	0.55969(5)
P1	4e	0.38948(13)	0.15524(6)	0.31632(13)
P2	4e	0.83892(13)	0.27521(6)	0.25492(12)
P3	4e	0.26030(13)	0.48802(6)	0.34341(14)

Table S4. Anisotropic displacement parameters ($Å^2$) for $Na_3Ge_2P_3$ from a single crystal structure determination at 298 K.

Atom	U_{11}	U_{22}	U_{33}	U_{12}	U_{13}	U_{23}
Na1	0.0332(9)	0.0240(8)	0.0333(9)	-0.0033(7)	0.0186(8)	-0.0019(7)
Na2	0.0209(8)	0.0206(8)	0.0281(8)	0.0018(6)	0.0015(6)	-0.0054(6)
Na3	0.0658(17)	0.0231(10)	0.0683(15)	0.0002(10)	-0.0146(13)	0.0041(10)
Ge1	0.01251(15)	0.00985(15)	0.01004(14)	0.00027(12)	0.00470(11)	-0.00035(12)
Ge2	0.01399(16)	0.00874(15)	0.01314(15)	0.00093(12)	0.00567(12)	0.00023(12)
P1	0.0147(4)	0.0160(4)	0.0160(4)	-0.0028(3)	0.0078(3)	-0.0032(3)
P2	0.0130(4)	0.0196(4)	0.0113(4)	-0.0032(3)	0.0053(3)	-0.0029(3)
P3	0.0150(4)	0.0115(4)	0.0206(4)	-0.0001(3)	0.0035(3)	0.0010(3)

Table S5. Selected interatomic distances in $Na_3Ge_2P_3$ from a single crystal structure determination at 298 K.

Atoms	Distance [Å]	Atoms	Distance [Å]
Ge1—Ge2	2.4885(5)	P1—Na2^{iii}	2.8726(18)
		P1—Na1^{i}	2.9203(19)
Ge1—P1	2.3405(10)	P1—Na3	2.996(2)
Ge1—P2	2.3191(9)	P1—Na1	3.044(2)
Ge1—P2^{i}	2.3238(10)	P1—Na3^{iv}	3.194(2)
Ge2—P1^{i}	2.3273(9)	P2—Na2^{v}	2.8414(17)
Ge2—P3	2.3577(10)	P2—Na3^{iii}	2.969(2)
Ge2—P3^{ii}	2.3607(10)	P2—Na1^{iv}	3.0000(19)
		P2—Na2^{vi}	3.1905(19)
		P2—Na1^{vii}	3.267(2)
		P3—Na2^{viii}	2.8482(19)
		P3—Na1	2.9646(19)
		P3—Na2	3.1016(19)
		P3—Na3^{ix}	3.150(3)

Symmetry operations: (i) x, $-y+{}^{1}/_{2}$, $z+{}^{1}/_{2}$; (ii) $-x+1$, $-y+1$, $-z+1$; (iii) x, $-y+{}^{1}/_{2}$, $z-{}^{1}/_{2}$; (iv) $-x+1$, $-y$, $-z+1$; (v) $x+1$, y, z; (vi) $x+1$, $-y+{}^{1}/_{2}$, $z-{}^{1}/_{2}$; (vii) $x+1$, $-y+{}^{1}/_{2}$, $z+{}^{1}/_{2}$; (viii) $-x$, $-y+1$, $-z+1$; (ix) $-x+1$, $y+{}^{1}/_{2}$, $-z+{}^{1}/_{2}$.

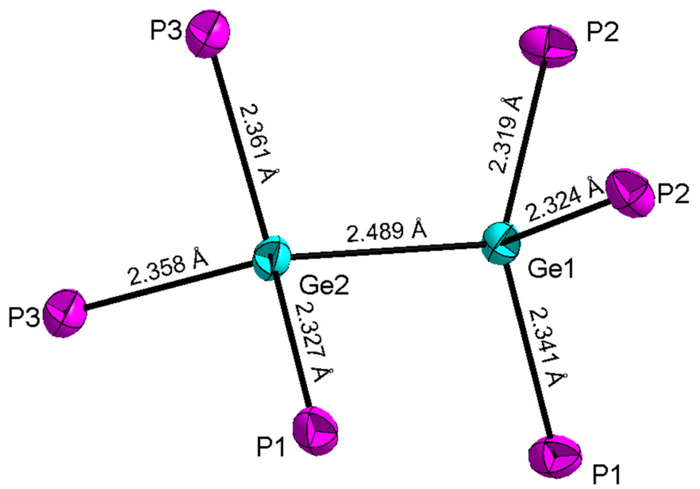

Figure S3. Dimeric [P_3Ge-GeP_3] building unit in $Na_3Ge_2P_3$, consisting of two interpenetrated Ge(GeP_3) tetrahedra. Bond lengths and anisotropic displacement ellipsoids are obtained from single crystal structure determination at room temperature, ellipsoids are drawn with 70 % probability level.

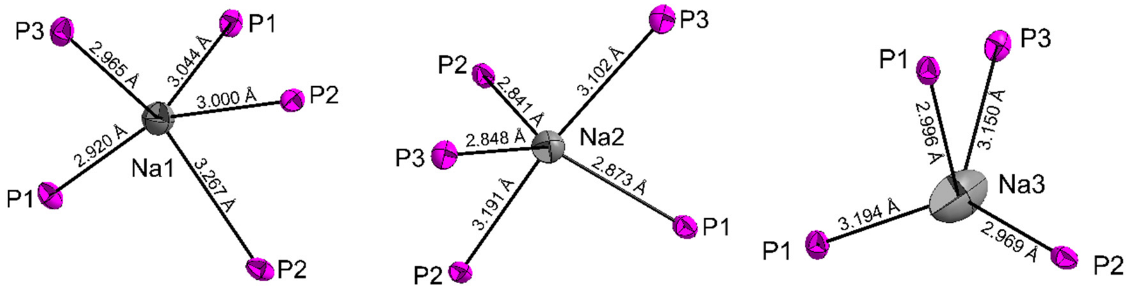

Figure S4. Coordination polyhedra of the three independent Na atoms in $Na_3Ge_2P_3$. For Na1 and Na2 the phosphorous neighbors form distorted trigonal bipyramids while for Na3 a strongly distorted tetrahedron is observed. Bond lengths and anisotropic displacement ellipsoids are obtained from single crystal structure determination at room temperature, ellipsoids are drawn at the 70 % probability level.

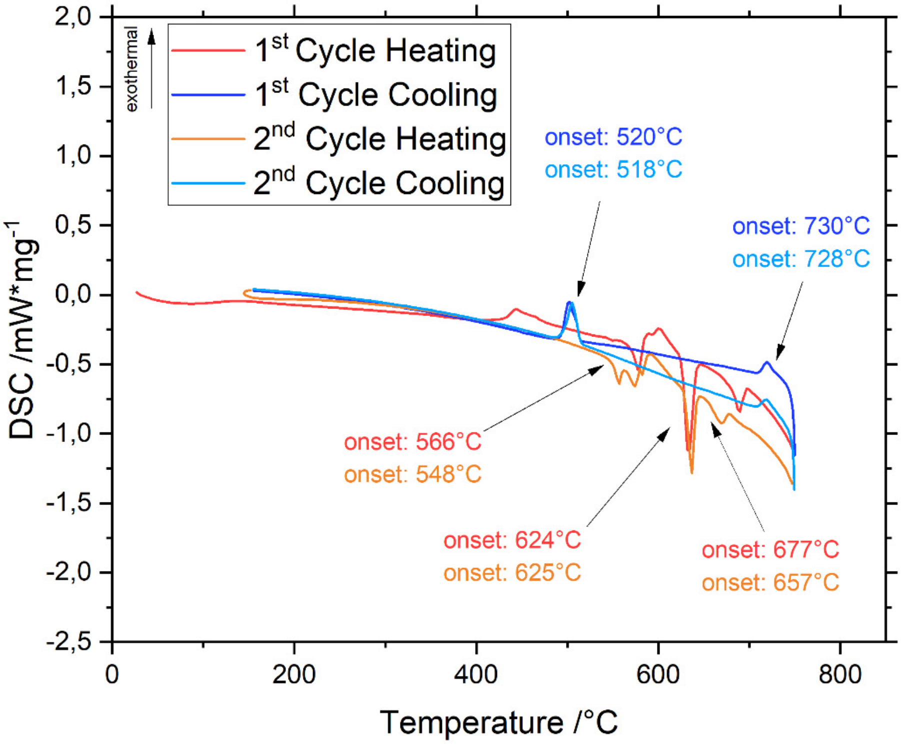

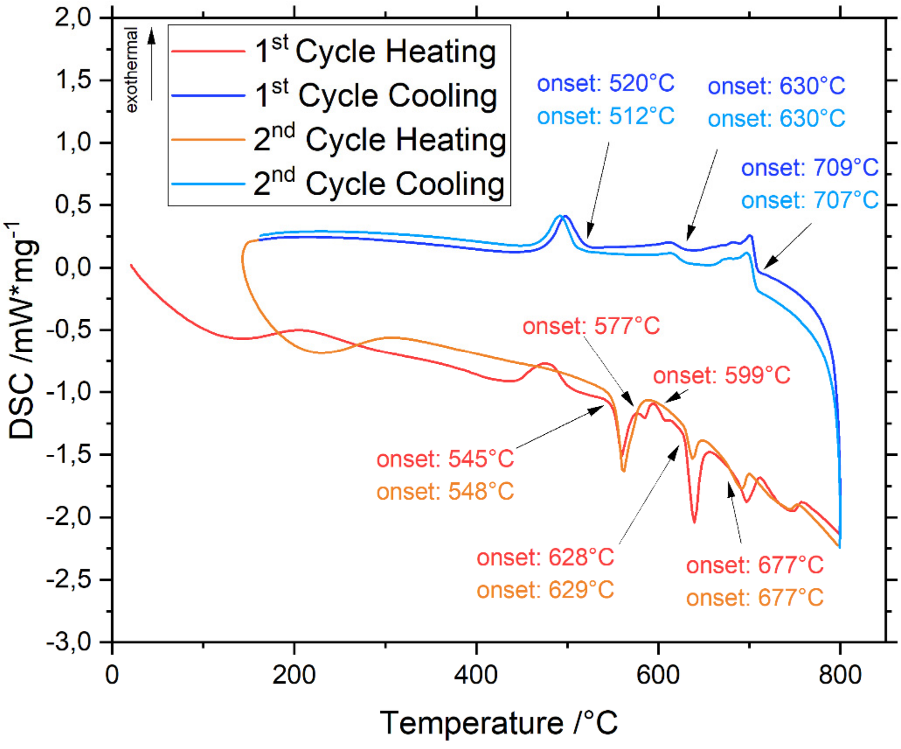

Figure S5. DSC curves of the reactive mixture after ball milling of "$Na_3Ge_2P_3$" (lower graph) and after annealing of the reactive mixture (upper graph). Two measurement cycles were performed using heating/cooling rates of 10 K·min^{-1}.

DSC measurements of $Na_3Ge_2P_3$ samples before and after annealing process reveal several phase transitions at roughly 545°C, 628°C and 675°C as well as a phase formation at 577°C. Comparing these finding to DSC evaluation performed for the phases $Na_2Ge_3P_3$ and $Na_5Ge_7P_5$, the signal at 545°C can be attributed to the formation of $Na_2Ge_3P_3$.[6] This is further confirmed by experiments on the synthesis of $Na_3Ge_2P_3$ at a temperature of 550°C. The phase formation at 577°C can be attributed to the formation of $Na_3Ge_2P_3$. Further heating seemingly induces decomposition of the phases as X-ray diffraction analysis of the DSC samples after measurement have resulted in a mainly amorphous diffractogram that only shows reflexes that cannot be assigned to any known phase. A Similar trend is observed for the synthesis of $Na_2Ge_3P_3$. [6]

Details of the DFT-PBE0 quantum chemical calculations

Table S6. Experimental and DFT-PBE0 interatomic distances and cell parameters for $Na_3Ge_2P_3$ as well as the deviation (Δ*d*) from experimental data in percentages.

Parameter	Calc.	Exp.	Deviation [%]
a	7.2136 Å	7.2894 Å	1.05
b	14.6653 Å	14.7725 Å	0.73
c	7.0381 Å	7.0528 Å	0.20
β	107.257°	106.331°	0.86
Ge1-P2	2.3183 Å	2.3191 Å	0.03
Ge1-P2’	2.3222 Å	2.3238 Å	0.07
Ge1-P1	2.3407 Å	2.3406 Å	0.00
Ge1-Ge2	2.4823 Å	2.4885 Å	0.25
Ge2-P1	2.3280 Å	2.3273 Å	0.03
Ge2-P3	2.3518 Å	2.3577 Å	0.25
Ge2-P3’	2.3613 Å	2.3607 Å	0.02

Table S7. Detailed description of the used basis sets and basis set listings in CRYSTAL format.

Na: Taken from reference [1].

```
11 5
0 0 5 2.0 1.0
  4098.2003908     -.58535911879E-02
  616.49374031     -.43647161872E-01
  139.96644001     -.19431465884
  39.073441051     -.48685065731
  11.929847205     -.41881705137
0 0 3 2.0 1.0
  20.659966030      .85949689854E-01
  1.9838860978     -.56359144041
  .64836323942     -.51954009048
0 0 1 1.0 1.0
  0.32              1.0000000000
0 1 1 0.0 1.0
  0.16              1.0 1.0
0 2 5 6.0 1.0
  75.401862017     .154353625324E-01
  17.274818978     .997382931840E-01
  5.1842347425     .312095939659
  1.6601211973     .492956748074
  .51232528958     .324203983180
```

P: Taken from reference [2].

```
15 8
0 0 7 2.0 1.0
  52426.999233       0.55207164100E-03
  7863.2660552       0.42678595308E-02
  1789.5227333       0.21931529186E-01
  506.27300165       0.85667168373E-01
  164.60698546       0.24840686605
  58.391918722       0.46336753971
  21.643663201       0.35350558156
0 0 3 2.0 1.0
  99.013837620       0.21895679958E-01
  30.550439817       0.95650470295E-01
  5.4537087661      -0.29454270186
0 0 2 2.0 1.0
  2.6477257457        1.3294381200
  1.2738231734       0.66109396473
0 1 1 0.0 1.0
  0.35000000000      1.0 1.0
0 1 1 0.0 1.0
  0.13000000000      1.0 1.0
0 2 6 6.0 1.0
  472.27219248       0.25710623052E-02
  111.58882756       0.20250297999E-01
  35.445936418       0.91580716787E-01
  12.990776875       0.25749454014
  5.0486221658       0.42862899758
  1.9934049566       0.34359817849
0 2 1 3.0 1.0
 0.69644412108       1.00000000000
0 3 1 0.0 1.0
  0.450              1.0
```

Ge: Taken from reference [3].

```
32 14
0 0 8 2.0 1.0
  466115.00592       0.22487264660E-03
  69875.420762       0.17435426729E-02
  15903.276716       0.90691482206E-02
  4501.8233453       0.36906174685E-01
  1466.0570924       0.12050167907
  527.07841728       0.28748641703
  205.00395074       0.41622321885
  81.251596065       0.22397845695
0 0 4 2.0 1.0
  505.74661282      -0.25184609291E-01
  156.96593744      -0.11898929721
  25.761448176       0.54930135870
  11.106654687       0.52939309129
0 0 2 2.0 1.0
  17.272059104      -0.22854595728
  2.9438289048       0.68377930317
0 0 1 2.0 1.0
  1.2852272293        1.0000000000
0 0 1 0.0 1.0
 0.30110831584        1.0000000000
0 1 1 0.0 1.0
 0.11000000000        1.0 1.0
0 2 6 6.0 1.0
  2633.9346241       0.22143925310E-02
  624.00161628       0.18140899141E-01
  200.58528404       0.86632184922E-01
  75.097081525       0.25649020592
  30.214388474       0.42658611262
  12.440087567       0.26200527313
0 2 3 6.0 1.0
  45.981316002      -0.20321767678E-01
  6.9945654416       0.32013744527
  2.9686001327       0.59051014555
0 2 1 2.0 1.0
 1.2452540491         1.0000000000
0 2 1 0.0 1.0
 0.36766682938        1.0000000000
0 3 5 10.0 1.0
  122.93850231       0.10586544521E-01
```

```
  36.242755203      0.69601280945E-01
  13.191062921      0.22807035287
  5.2163136729      0.40301067220
  2.0927838749      0.41304847015
0 3 1 0.0 1.0
 0.81259117944       1.0000000000
0 3 1 0.0 1.0
 0.24600000000       1.0000000000
0 4 1 0.0 1.0
 0.36210645          1.0
```

Table S8. Reciprocal space coordinates for the band path used for the primitive monoclinic lattice. The Brillouin zone path Γ-Z-D-B-Γ-A-E-Z-C_2-Y_2-Γ is derived from the SeeK-path web service [4-5].

	×$\boldsymbol{b_1}$	×$\boldsymbol{b_2}$	×$\boldsymbol{b_3}$
Γ	0	0	0
Z	0	-½	0
D	0	½	½
B	0	0	½
A	-½	0	½
E	-½	½	½
C_2	-½	½	0
Y_2	-½	0	0

Table S9. CIF details of structure optimization.

```
data_findsym-output

_audit_creation_method FINDSYM


_cell_length_a    7.2136728300

_cell_length_b    14.6653771100

_cell_length_c    7.0381267300

_cell_angle_alpha 90.0000000000

_cell_angle_beta  107.2578460000

_cell_angle_gamma 90.0000000000


_symmetry_space_group_name_H-M "P 1 21/c 1"

_symmetry_Int_Tables_number 14

_space_group.reference_setting '014:-P 2ybc'

_space_group.transform_Pp_abc a,b,c;0,0,0


loop_

_space_group_symop_id

_space_group_symop_operation_xyz

1 x,y,z

2 -x,y+1/2,-z+1/2

3 -x,-y,-z

4 x,-y+1/2,z+1/2


loop_

_atom_site_label

_atom_site_type_symbol

_atom_site_symmetry_multiplicity
```

```
_atom_site_Wyckoff_label
_atom_site_fract_x
_atom_site_fract_y
_atom_site_fract_z
_atom_site_occupancy
Na1 Na   4 e 0.17421 0.81787  0.59802  1.00000
Na2 Na   4 e 0.01145 0.89316  0.06948  1.00000
Na3 Na   4 e 0.71147 0.54580  0.06819  1.00000
Ge1 Ge   4 e 0.63063 0.76124  -0.05333 1.00000
Ge2 Ge   4 e 0.49872 -0.09883 0.05724  1.00000
P1  P    4 e 0.38670 0.65381  0.81345  1.00000
P2  P    4 e 0.84182 0.77574  0.75550  1.00000
P3  P    4 e 0.25509 -0.01205 0.83452  1.00000
```

References:

[1] R. E. Stene, B. Scheibe, A. J. Karttunen, W. Petry, F. Kraus, *Eur. J. Inorg. Chem.* **2019**, *2019*, 3672-3682.

[2] G. Sansone, L. Maschio, D. Usvyat, M. Schütz, A. Karttunen, *J. Phys. Chem. Lett.* **2016**, *7*, 131-136.

[3] L. M. Scherf, A. J. Karttunen, O. Pecher, P. C. M. M. Magusin, C. P. Grey, T. F. Fässler, *Angew. Chem. Int. Ed.* **2016**, *55*, 1075-1079.

[4] Y. Hinuma, G. Pizzi, Y. Kumagai, F. Oba, I. Tanaka, *Comput. Mater. Sci.* **2017**, *128*, 140-184.

[5] A. H. Larsen, J. J. Mortensen, J. Blomqvist, I. E. Castelli, R. Christensen, M. Dułak, J. Friis, M. N. Groves, B. Hammer, C. Hargus, E. D. Hermes, P. C. Jennings, P. B. Jensen, J. Kermode, J. R. Kitchin, E. L. Kolsbjerg, J. Kubal, K. Kaasbjerg, S. Lysgaard, J. B. Maronsson, T. Maxson, T. Olsen, L. Pastewka, A. Peterson, C. Rostgaard, J. Schiøtz, O. Schütt, M. Strange, K. S. Thygesen, T. Vegge, L. Vilhelmsen, M. Walter, Z. Zeng, K. W. Jacobsen, *J. Phys.: Condens. Matter* **2017**, *29*, 273002.

[6] H. Eickhoff, V. Hlukhyy, T. F. Fässler, *Z. Anorg. Allg. Chem.* **2020**, *646* (22), 1834-1838.
